# CMHT autonomous dataset: A multi-sensor dataset including radar and IR for autonomous driving

**DOI:** 10.1016/j.dib.2025.111552

**Published:** 2025-04-11

**Authors:** Howard Zhang, Ash Liu, Saied Habibi, Martin v. Mohrenschildt, Ryan Ahmed

**Affiliations:** a Department of Computing and Software, McMaster University 1280 Main St. W, Hamilton, ON, L8S 4L8, Canada; b Center for Mechatronics and Hybrid Technologies (CMHT), Department of Mechanical Engineering, 200 Longwood Road South, Unit 224, Hamilton, ON, L8P0A6, Canada

**Keywords:** Autonomous driving, Sensor fusion, LiDAR, Radar, Infrared camera, GPS/IMU

## Abstract

Standardized datasets are essential for the development and evaluation of autonomous driving algorithms. As the types of sensors available to researchers increase, datasets containing a variety of temporally and spatially aligned sensors have become increasingly valuable. This paper presents a driving dataset recorded using a complete sensor suite for research on autonomous driving, perception, and sensor fusion. The dataset consists of over 9000 frames of data recorded at 10-20Hz using a complete sensor suite made up of Velodyne LiDAR, GPS/IMU, mm-wave radar, as well as color and infrared cameras. The capture scenarios include poor weather/lighting conditions, such as rain/night scenarios, and diverse traffic conditions, such as highways and cities with various objects. Both fully synchronized data and raw recordings in the form of ROS2 bags are provided, as well as 3D tracklet labels for individual objects. This paper provides technical details on the driving platform, data format, and utilities.

Specifications Table

**Table:** 

Subject	Signal Processing Computer Vision and Pattern Recognition
Specific subject area	Multimodal Dataset for Autonomous driving
Type of data	Raw: ros2bags .pcd(lidar & radar) .png(camera & IR camera) .txt(GPS/IMU) .JSON(Labels & calibration files)
Data collection	Data was collected using a vehicular sensor platform. The raw data was recorded using ROS2 bags and later extracted to a frame by frame structure. The extracted frames is then labelled using SUSTECHPOINTS. hardware: velodyne HDL-32E Lidar FLIR A65 thermal camera Retina-4fn mmWave radar Logitech Brio monocular camera
Data source location	Data is collected around the city of Hamilton, Ontario, Canada Data is stored in MacDrive by McMaster University
Data accessibility	Repository name: Federated Research Data Repository (FRDR) Direct URL to data:https://macdrive.mcmaster.ca/d/2d54f23bc41f48bd9a2d/ https://doi.org/10.20383/103.01024
Related research article	none

## 1 Value of the Data

• This dataset is useful to further develop algorithms and techniques to the field of autonomous driving. The different sensors can be both combined and compared with one another to evaluate their efficacy in different weather conditions, and improve / develop existing / new models for high level autonomous systems.
• The Data is valuable due to the multi-modal sensor nature. Specifically the combination of Radar and IR along with the more common lidar and camera is first of its kind (from our search) as well as the variety of weather / lighting conditions present in the dataset.
• The Data can be used by other researchers for a variety of tracking, computer-vision, and sensor fusion applications by comparing their results to our labelled ground truths. The labelled data can also be used in AI training applications

## 2 Background

The field of autonomous driving is based on the foundation of heterogeneous sensors and techniques that combine these sensors in sensor fusion to achieve both robust and accurate performance. Therefore, the use of datasets to train and evaluate machine learning models and classical methods has become the norm in this field. However, a key issue in the datasets available is the types of sensors available. For example, both KITTI [1] and Waymo [3] only provide camera and LiDAR data, while NuScenes [2] additionally provides radar data. Research reported in [4,5], and [6] have shown that the efficacy of these sensors deteriorates in harsh weather/lighting conditions. Therefore, a dataset that provides auxiliary sensors that can compensate LiDAR and cameras such as radar and infrared cameras is necessary to explore sensor fusion in these conditions.

The Centre for Mechatronics and Hybrid Technologies (CMHT) research group at McMaster University works on various fields including signal processing [7] and target tracking [8]. The CMHT autonomous dataset is a natural extension to previous works and aims to further development in these fields.

The CMHT autonomous dataset consists of color and IR camera images, LiDAR point clouds, radar point clouds, and IMU/GPS measurements built into the LiDAR. The purpose of this data set is to contribute meaningful data to further the development of autonomous driving and sensor fusion. This paper includes technical details on the sensor setup, the data format, and instructions on accessing the dataset.

## 3 Data Description

**Fig. 1:**
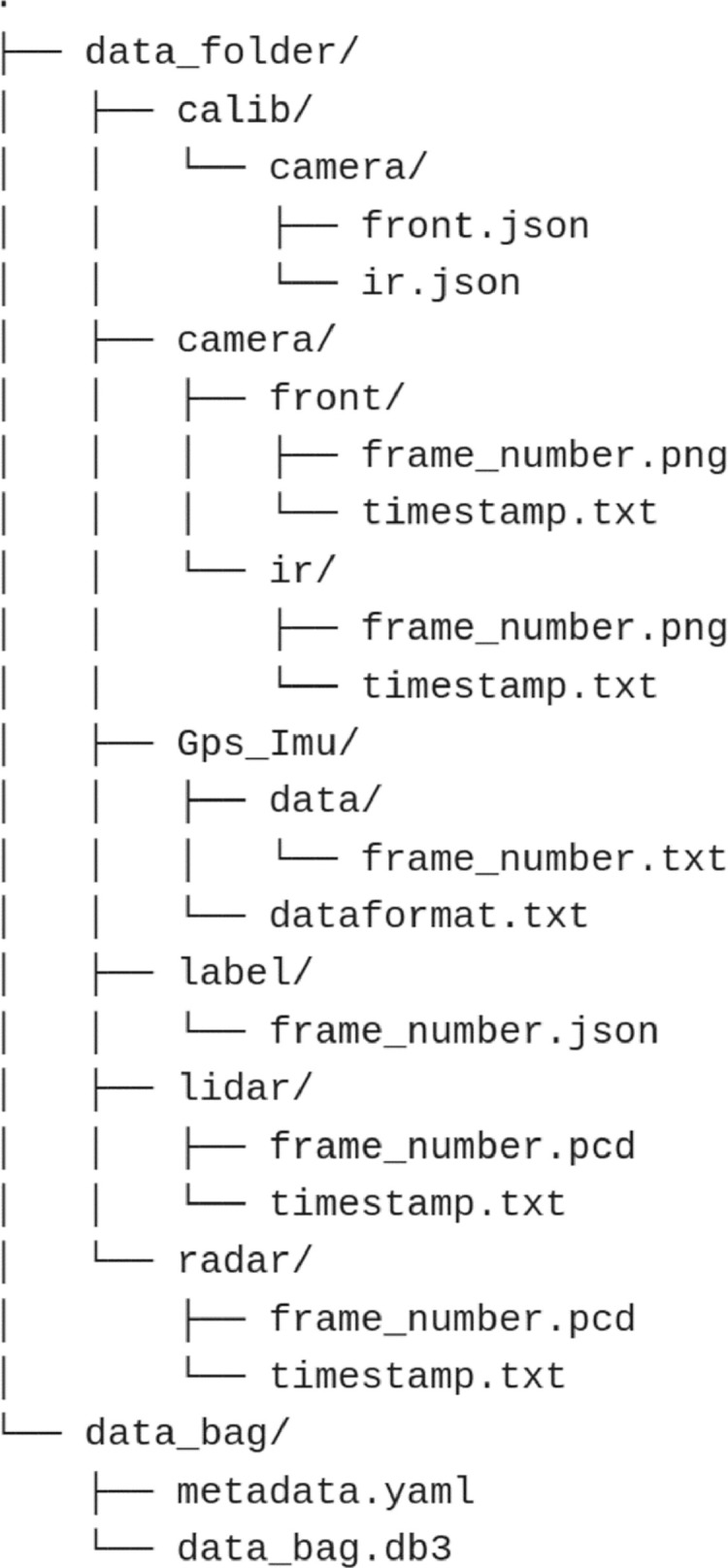
File structure of dataset.

Individual recordings are compressed into a .zip file named in the convention drive_lighting_weather_ID.zip. The file structure is shown in Fig. 1. The raw recording is compressed into a separate .zip file named drive_lighting_weather_ID_bag.zip, and can be read using ROS2 command tools. A ``timestamp.txt`` is provided in each individual sensor folder as the timestamp used by the synchronizer when all the sensor readings are collected.

• *Images:* The monocular camera frames are saved as 1280×720 RGB images, and the IR camera is saved as 640×480 greyscale images.
• *GPS/IMU:* The GPS/IMU data originates from the Velodyne LiDAR, consisting of 3 ADXL203 accelerometers, 3 ADIS16080 gyroscopes, and a GPS 18x LVC GPS receiver
• *Velodyne LiDAR:* The LiDAR point clouds are saved as standard .pcd files containing [x,y,z,i] fields and can be easily parsed with built-in functions provided by most tools such as Matlab or python.
• *Radar:* The radar point clouds are saved as a standard .pcd file containing [x,y,z,v] fields and can be simply parsed with the same method as the LiDAR point clouds.

In the data_folder, the calib folder contains both the intrinsic and extrinsic matrices used for bounding box projections for the IR and monocular cameras. The camera folder contains individual frames and timestamps of the frames for IR and monocular cameras in their respective folder. The GPS_Imu folder contains a txt file in the data subfolder that contains the GPS/IMU data of that frame, the dataformat.txt file explains how to parse said data. The label folder contains .json files for the 3D tracklet labels of objects in every frame. the lidar and radar folders both contain .pcd files for each frame and their respective timestamps in timestamp.txt

**Annotations:**

For a detected object by the LiDAR, the information provided includes a 3D bounding box, and a classification in the set ``Car``,``Truck``,``Van``,``Pedestrian``,``Bus``, and ``LongVehicle``.

The labels for each frame are stored in frame_number.json.

Every object is labelled with its 3D position, size, rotation, classification, and object ID. Note that while only the 3D tracklets are given, said labels can be projected onto the other sensors for training and testing purposes.

**SDK:**

Two example scripts are provided in Matlab and Python that demonstrate working with the dataset.

The files CMHT_projection.m and CMHT_projection.py extracts tracklet information from a single frame and projects the bounding boxes onto both monocular and IR cameras.

## 4 Experimental Design, Materials and Methods

Robust and precise system is required to ensure data quality and spatial and temporal stability. This section describes the sensors and software built to record the dataset.

**Fig. 2:**
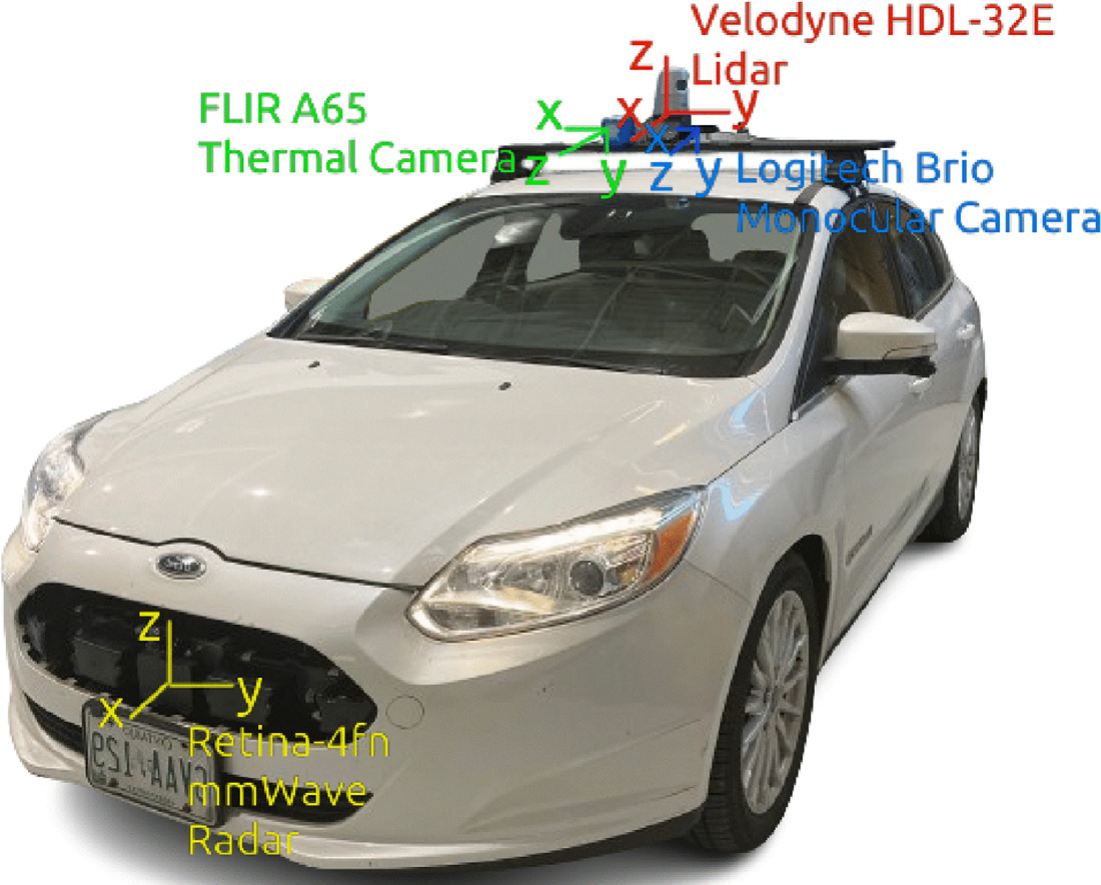
Recording vehicle.

The Sensor setup is shown in Fig. 2, and consists of the following sensors:

• 1 x Velodyne HDL-32E LiDAR, 10Hz, 32 channels, 100m ± 0.02m range, 41.33° ± 1.33° vertical FOV, 360° ± 0.1° horizontal FOV
• 1 x FLIR A65 thermal camera, 30Hz, 25° horizontal FOV, 20° vertical FOV, 640×512 resolution.
• 1 x Retina-4fn mmWave radar, 20Hz, range: 250m, 30° ± 15° horizontal FOV, 24° ± 12° vertical FOV
• 1 x Logitech Brio monocular camera, 30/60 FPS, 90° diagonal FOV, 13 Megapixels

**Fig. 3:**
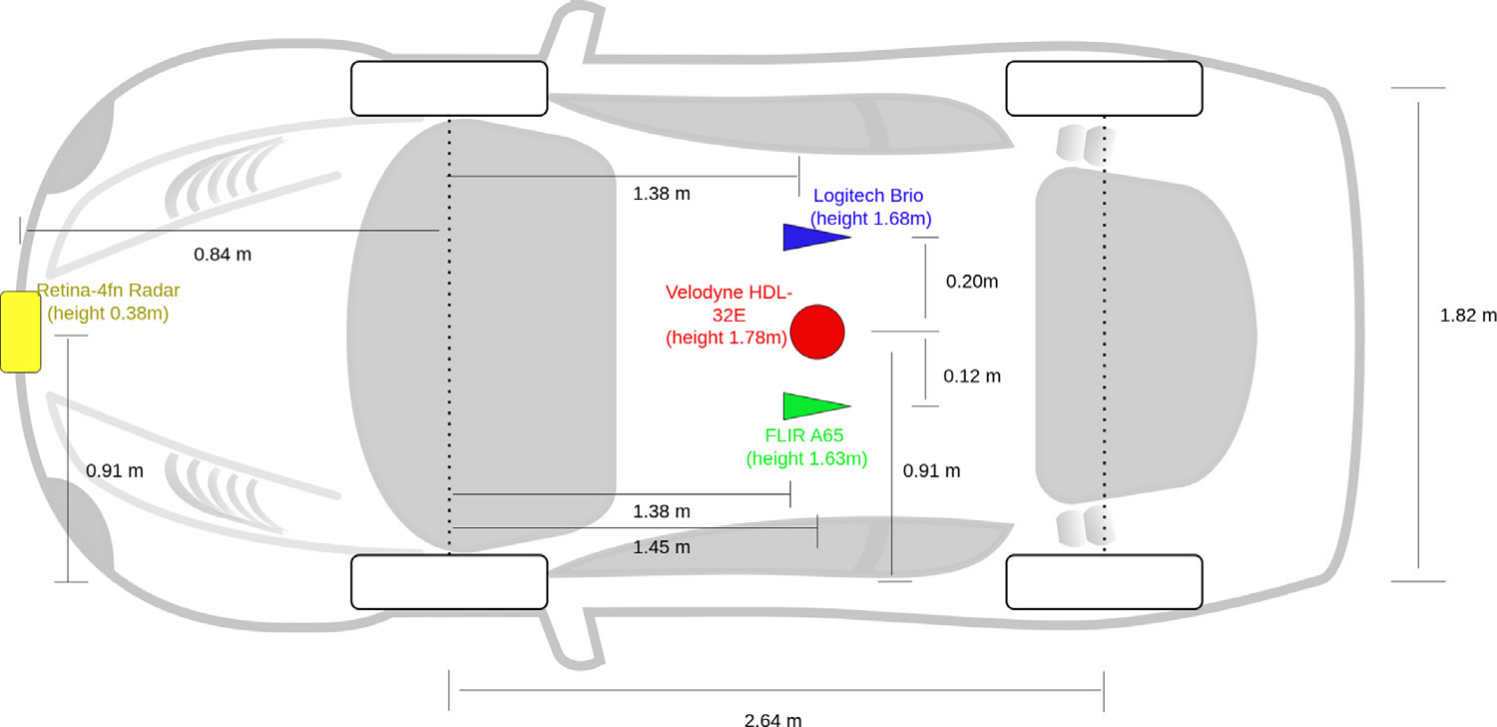
.Technical drawing of sensor layout. All heights are relative to the ground.

The sensor layout is shown in Fig. 3, and the system is controlled by a central laptop with a 14-core Intel i7-12800H processor running Ubuntu Linux (64-bit) and using a ROS2 [9] based recording software to record sensor data.

The recording software was built using ROS2-galactic. ROS2 is an open-source robotics middleware that uses a node-based architecture consisting of publishers and subscribers. Each node consists of a single sensor publisher using the sensor data qos profile, and the data is recorded using the ROS2 bag functionality. As ROS2 is designed to be real-time capable, the system is also designed to be used in real-time computing and communication. A latency and jitter test on the system was performed using the methodology in [10]. The test ran for 350 samples and showed an average delay of 99.83 ms, and a jitter of 0.000001% relative to the frame interval of 100 milliseconds. This high stability was achieved after tuning the DDS parameters according to [11]. The parameters used were cyclone dds, with rmem max=2147483647 and wmem max=12582912.

### 4.1 Sensor calibration

#### 4.1.1 Temporal calibration

The Velodyne LiDAR is the reference that triggers both monocular and IR cameras. The GPS/IMU and radar could not be triggered and so instead runs on their own, and is synchronized with the LiDAR via closest timestamp. The timestamps are all generated by the system clock on the host computer. The system first records all data using a ROS2 bag, and individual frames are extracted by matching timestamps using the ROS2 ApproximateTimeSynchronizer with a tolerance of 50ns.

#### 4.1.2 Camera calibration

Camera-LiDAR calibration involves calculating an intrinsic and extrinsic transformation matrix in order to map 3D LiDAR coordinates [x,y,z,1] to the 2D camera coordinates [u,v,1].

The method proposed in [12] was used to obtain both matrices using a checkerboard. The extrinsic matrix aligns the axis of the LiDAR to the axis of the camera, and the intrinsic matrix projects the resulting 3D spatial point into a 2D pixel point.

#### 4.1.3 IR Calibration

While the mathematical theory used in monocular also applies to IR cameras, practical differences in the image obtained by an IR camera compared to a regular camera make identifying features on the checkerboard difficult on a normal board. To solve this issue, the method developed by [13] was employed, and a special board with detachable black cells was heated to create enough contrast to use the same method as the regular camera.

#### 4.1.4 Radar-LiDAR alignment

As the radar and LiDAR use the same 3D coordinate system, only an extrinsic transformation matrix is needed. Due to the rigid fixture of the LiDAR and radar, the translation matrix was tuned by comparing points generated from the same object on both sensors and calculating the difference. The two-point clouds have been aligned at the recording level for ease of use. The rotation matrix is neglected after checking that the point clouds post translation had minuscule deviation, which means rotation is unnecessary.

## Limitations

Some limitations of this dataset include:

• Low resolution lidar: compared to newer datasets, the 32 channel lidar is outdated in terms of lidar resolution
• Size: due to the limitations of manual labelling, the amount of frames provided by this dataset is below that of the more popular autonomous driving datasets
• FOV: the current sensor setup means that overlapping FOV between all sensors only exist at the front of the vehicle, which limits sensor fusion to this specific FOV
• low pedestrian data: The current dataset has a low amount of pedestrians in its recording, which may cause detrimental effects in AI training/testing.

## Ethics Statement

The authors have read the ethical requirements and confirm that this dataset does not involve any human subjects, animal experiments, or data from social media platforms.

## CRediT authorship contribution statement

**Howard Zhang:** Conceptualization, Methodology, Data curation, Writing – original draft. **Ash Liu:** Conceptualization, Methodology, Data curation. **Saied Habibi:** Conceptualization, Resources, Supervision, Writing – review & editing. **Martin v. Mohrenschildt:** Supervision, Writing – review & editing. **Ryan Ahmed:** Supervision, Writing – review & editing.

## Data Availability

MacDrive by McMaster UniversityCMHT Autonomous Dataset (Original data)
Federated Research Data RepositoryCMHT Dataset: Driving dataset containing labeled and synchronized Lidar, Radar, IR, Camera, GPS/IMU recordings (Original data)
